# CircHN1 affects cell proliferation and migration in gastric cancer

**DOI:** 10.1002/jcla.23433

**Published:** 2020-07-01

**Authors:** Yu Zhang, Maoye Wang, Xueyan Zang, Zheying Mao, Yanke Chen, Fei Mao, Hui Qian, Wenrong Xu, Xu Zhang

**Affiliations:** ^1^ Jiangsu Key Laboratory of Medical Science and Laboratory Medicine School of Medicine Jiangsu University Zhenjiang Jiangsu China; ^2^ Key Laboratory of Molecular Diagnostics and Precision Medicine for Surgical Oncology in Gansu Province Gansu Provincial Hospital Gansu China

**Keywords:** circHN1, circular RNA, gastric cancer, migration, proliferation

## Abstract

**Background:**

Increasing evidence indicates that circular RNAs (circRNAs) are dysregulated in human cancers. The biological roles of circRNAs in gastric cancer (GC) have not been well‐characterized.

**Methods:**

The GEO database was used to analyze circRNA expression profile in GC. The expression level of target circRNA in tumor tissues and adjacent non‐tumor tissues was detected by reverse transcription‐quantitative PCR. Gene transfection was used to manipulate the expression of circRNAs. The biological roles of circRNAs in cell proliferation, migration, and invasion were determined by cell counting, colony formation, transwell migration, Matrigel invasion, and mouse xenograft tumor assays. The interactions between circRNAs and miRNAs were verified by RNA immunoprecipitation and luciferase reporter assays.

**Results:**

We found that circHN1 was upregulated in GC tissues and cell lines compared to adjacent non‐tumor tissues and normal gastric epithelial cells. Additionally, circHN1 silencing significantly promoted GC cell growth, colony formation, migration, and invasion, whereas circHN1 overexpression had the opposite effects. CircHN1 overexpression also suppressed gastric cancer growth in the mouse xenograft tumor model. CircHN1 was mainly localized in the cytoplasm of GC cells and could bind to AGO2. MiR‐1248 and miR‐375 were predicted to interact with circHN1 by bioinformatic analyses. MiR‐1248 and miR‐375 overexpression inhibited the activity of the circHN1 luciferase reporter.

**Conclusion:**

CircHN1 is aberrantly expressed in GC and affects the proliferation and migration of gastric cancer cells by acting as miRNA sponge.

## 1 INTRODUCTION

Gastric cancer (GC) is one of the leading causes of cancer‐related deaths.^1^ Despite recent improvements in detection and treatment, the morbidity and mortality of GC remain high.^2^ Therefore, new biomarkers and targets for gastric cancer diagnosis and treatment are urgently needed.

Circular RNAs (circRNAs) are endogenous RNAs produced by back‐splicing and have neither a polyadenylated tail nor a 5′ to 3′ polarity.^3^, ^4^ With the development of next‐generation sequencing and bioinformatics methods, many circRNAs have been shown to be involved in the pathogenesis of various diseases such as diabetes, heart diseases, psychiatric diseases, and cancers.^5^, ^6^, ^7^, ^8^

CircRNAs contain abundant miRNA binding sites.^9^ Recent evidence has indicated that circRNAs can serve as efficient miRNA sponges.^10^, ^11^, ^12^, ^13^ For instance, circRNA PRMT5 has been documented to function as miR‐30c sponge to induce epithelial‐mesenchymal transition in urothelial bladder carcinoma.^14^ In addition, circRNA MTO1 can sponge miR‐9 to inhibit the progression of hepatocellular carcinoma.^15^ However, the role and molecular mechanisms of circRNAs in GC are not fully understood.

In this study, we first studied the circRNA profile of GC in a circRNA database and focused on a new circRNA, circHN1. We then evaluated the expression pattern of circHN1 in GC tissues and investigated the biological roles of circHN1. Moreover, we investigated whether circHN1 could act as miRNA sponge in GC.

## 2 MATERIALS AND METHODS

### 2.1 Analysis of microarray data

The GSE83521 human circRNA microarray dataset was downloaded from the GEO database (http://www.ncbi.nlm.nih.gov/geo/). The transcriptome data of 6 pairs of gastric tumor tissues and non‐tumor tissues were included in the GSE83521 dataset. Quantile normalization and subsequent data processing were performed using the R software package.

### 2.2 Patients and tissue samples

Paired GC tissues and adjacent non‐tumor tissues (n = 101) were obtained from the Department of General Surgery, the Affiliated People's Hospital of Jiangsu University between June 2016 and April 2017. This study was reviewed and approved by the Jiangsu University Institutional Ethical Committee. Informed consent was obtained from all participants before sample collection.

### 2.3 Reverse transcription‐quantitative polymerase chain reaction (RT‐qPCR)

The HiScript 1st Strand cDNA Synthesis Kit (Vazyme) was used to reversely transcribe the RNA into cDNA. The relative expression levels of target genes were normalized to that of GAPDH. The sequences of circHN1 divergent primers were as follows: forward, 5′‐GCAGGTGCCAAGTCTAGTGG‐3′; reverse, 5′‐GCCGCAAAACTCATGAATATCACC‐3′. The sequences of GAPDH convergent primers were as follows: forward, 5′‐GGATTTGGTCGTATTGGG‐3′; reverse, 5′ ‐GGAAGATGGTGATGGGATT‐3′.

### 2.4 Cell culture and cell transfection

The human GC cell line MGC‐803 and gastric mucosa cell line GES‐1 were acquired from the Institute of Basic Medical Sciences, Chinese Academy of Medical Sciences (Beijing, China). Human GC cell line HGC‐27 was obtained from Cellcook Biotech. LipoFiter (Hanbio) was used to transfect overexpressing plasmid and knockdown shRNA (GenePharma) into the cells. The sequence of sh‐circHN1‐1 was 5′‐GAAGGTGATATTCATGAGTTT‐3′, and the sequence of sh‐circHN1‐2 was 5′‐GATATTCATGAGTTTTGCGGC‐3′.

### 2.5 Cell counting and cell colony formation assays

For cell counting assay, 1 × 10^4^ cells were seeded into each well of a 24‐well plate and counted every day. Cell growth curves were plotted using the results. For colony formation assay, 1 × 10^3^ cells were seeded into each well of a 6‐well plate. After ten days, the colonies were fixed with 4% paraformaldehyde, stained with violet crystal, and counted under a microscope.

### 2.6 Transwell migration and Matrigel invasion assays

Transwell migration and Matrigel invasion assays were conducted in transwell chambers (Corning, Inc). Serum‐free media was added to the top chamber, and complete medium was added to the lower chamber. In the transwell migration assay, 1 × 10^5^ cells were placed in the top chamber. The cells that had migrated to the other side of the membrane were counted after 24 hours. Matrigel was added to the top chamber for the Matrigel invasion assay. Next, 2 × 10^5^ cells were placed in the top chamber and incubated for 36 hours. The cells that had invaded to the other side of the membrane were counted.

### 2.7 RNA immunoprecipitation (RIP)

A Magna RIP kit (Millipore) was used to conduct the RIP assay. The cells were lysed, and the cell lysate was immunoprecipitated on magnetic beads by human anti‐AGO2 antibodies. An IgG antibody was used as the control. To detect RNA directly binding to AGO2, the immunoprecipitated RNA was purified and quantified by qRT‐PCR.

### 2.8 Luciferase reporter assay

CircInteractome (https://circinteractome.nia.nih.gov/index.html) and StarBase v2.0 (http://starbase.sysu.edu.cn) were used to predict the potential binding miRNAs. 293T cells (1.5 × 10^5^ cells/well) were co‐transfected with 0.5 μg of pGL3‐Luc‐circHN1 and 100 nmol miRNA mimics. The cells were lysed after 36 hours of transfection, and luciferase activity was analyzed by the Reporter Assay Program Dual‐Luciferase (Promega).

### 2.9 Animal studies

The animal study was approved by the Animal Use and Care Committee of Jiangsu University. Four‐week‐old male BALB/c nude mice were subcutaneously injected with MGC‐803 cells transfected with circHN1 or control vector. Four weeks later, the mice were sacrificed, and the tumors were harvested.

### 2.10 Statistical analyses

Data were analyzed using SPSS 20.0 software (SPSS Inc) and GraphPad Prism 7.0 software (GraphPad Inc). The expression level of circRNA was calculated as the ΔCt (Ct value of target −Ct value of GADPH). Fold changes were calculated using the 2^−ΔΔCt^ method. Data were compared between two groups by Student's *t* test or Wilcoxon rank‐sum test. A *P* value less than .05 was considered as statistically significant.

## 3 RESULTS

### 3.1 CircHN1 is upregulated in GC

**FIGURE 1:**
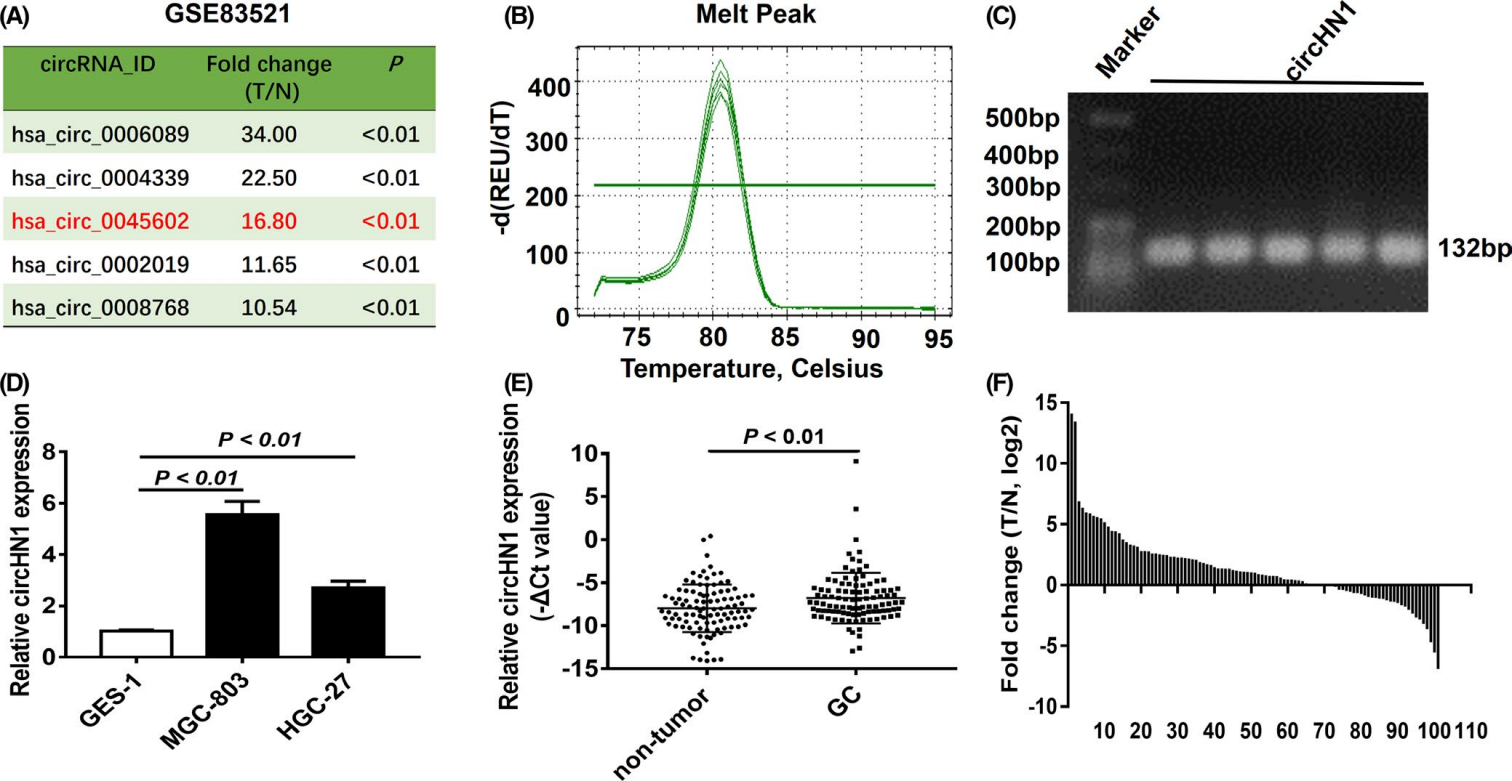
Expression of circRNA in GC. (A) Top 5 upregulated circRNAs in GSE83521 dataset. (B) The melting curve of qRT‐PCR for circHN1. (C) Agarose gel electrophoresis for circHN1 PCR products. (D) CircHN1 expression in GC cell lines was detected by qRT‐PCR. (E,F) CircHN1 expression in 101 paired GC tissues and non‐tumor tissues was detected by qRT‐PCR

We first downloaded the GSE83521 human circRNA microarray dataset from GEO and analyzed the differentially expressed circRNAs using the R software package. We selected the top 5 upregulated circRNAs (Figure 1A) according to the following criteria: fold change ≥2.0 and *P* value <.05. We next designed specific primers for qRT‐PCR detection. The melting curves of qRT‐PCR for hsa_circ_0006089, hsa_circ_0004339, and hsa_circ_0002019 did not show specific single peaks; however, the primer melting curves of qRT‐PCR for hsa_circ_0045602 and hsa_circ_0008768 showed single peaks, indicating that the primers for these two circRNAs were specific. Given that the fold change of hsa_circ_0045602 between GC and adjacent non‐tumor tissues in the microarray was higher than that of hsa_circ_0008768, we selected hsa_circ_0045602 as our target circRNA. We further verified the PCR product of hsa_circ_0045602 by agarose gel electrophoresis (Figure 1B,C). We selected hsa_circ_0045602 for further study and termed it “circHN1” as it is derived from *HN1* gene. We then detected the expression of circHN1 in both GC cell lines and a cohort of 101 paired GC and adjacent non‐tumor tissues. The expression levels of circHN1 in HGC‐27 and MGC‐803 cells were significantly higher than that in GES‐1 cells (Figure 1D). Compared to that in non‐tumor tissues, circHN1 expression was substantially upregulated in GC tissues (Figure 1E,F). We also explored the correlation between circHN1 expression and the clinicopathological parameters of patients with GC. The results showed that circHN1 expression was correlated with tumor invasion (Table 1).

**TABLE 1:** Relationship of circHN1 expression levels (ΔCt) in GC tissues with clinicopathological factors of GC patients

Parameters	No. of patients	Mean ± SD	*P* value
Gender			
Male	75	6.1 ± 2.9	.510
Female	26	6.5 ± 3.0	
Age (y)			
<60	28	6.1 ± 2.9	.811
≥60	73	6.2 ± 2.9	
Tumor size (cm)			
≤5	46	6.3 ± 3.4	.776
>5	55	6.1 ± 2.4	
Differentiation			
Poor	61	6.0 ± 2.6	.610
Well‐moderate	40	6.3 ± 3.4	
Invasion			
T1‐T3	28	4.9 ± 3.6	.006
T4	73	6.7 ± 2.5	
Lymphatic metastasis			
N0	34	5.6 ± 3.7	.148
N1‐N3	67	6.5 ± 2.4	
Distant metastasis			
M0	98	6.2 ± 2.9	.384
M1	3	4.7 ± 1.0	
TNM stage			
I—II	40	5.7 ± 3.5	.200
III—IV	61	6.5 ± 2.5	
CA19‐9			
Negative	79	6.0 ± 3.0	.362
Positive	22	6.5 ± 2.8	
Nervous invasion			
Absent	84	5.9 ± 3.1	.001
Present	17	7.7 ± 1.3	
Perineural invasion			
Absent	85	5.9 ± 3.1	.003
Present	16	7.4 ± 1.1	

### 3.2 CircHN1 is generated from exons 3 to 5 of *HN1* by back‐splicing

**FIGURE 2:**
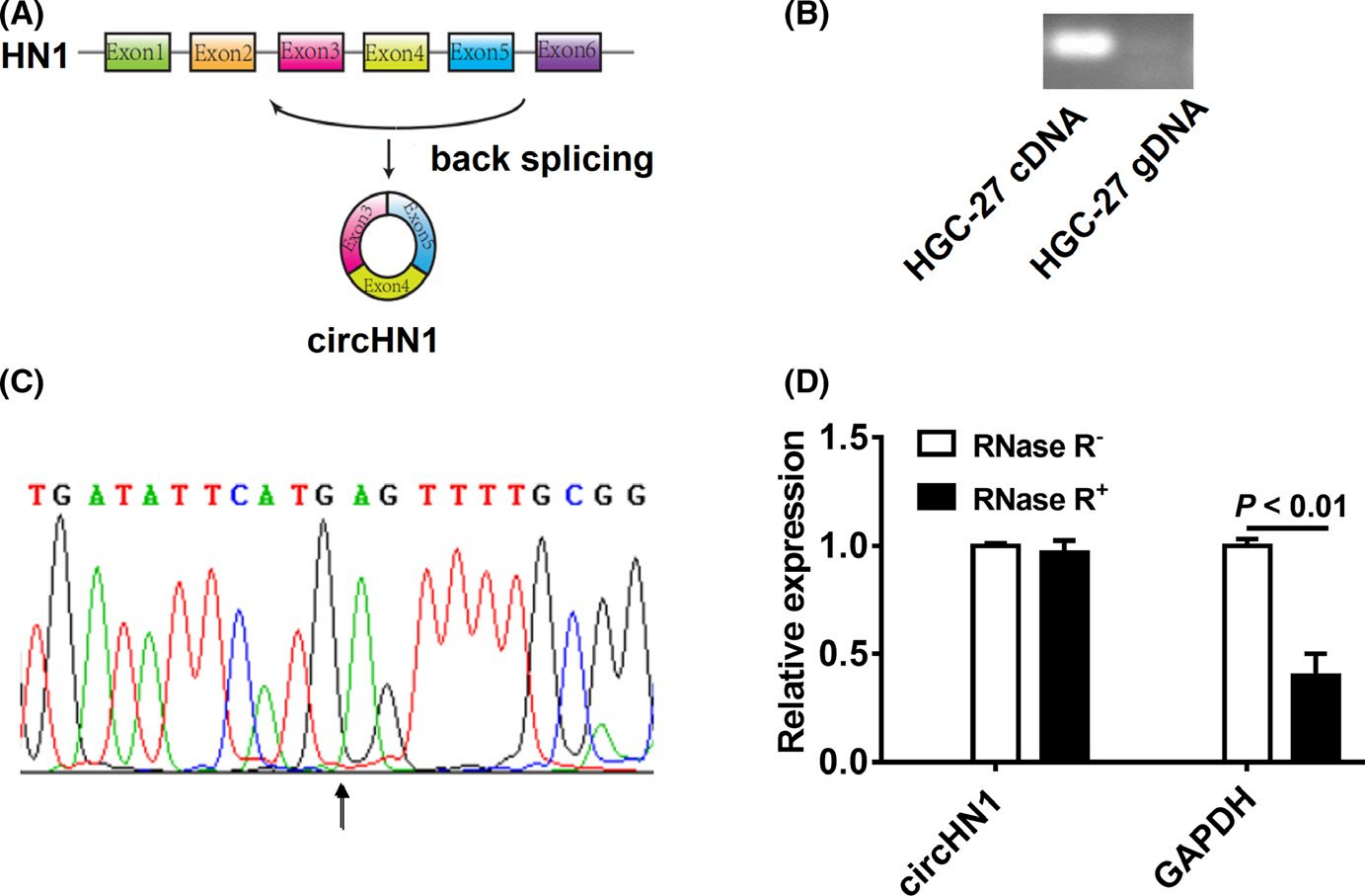
Identification of circHN1. (A) Schematic drawing of the genomic location of circHN1. (B) PCR amplification of circHN1 with divergent primer by using cDNA and gDNA as the templates. (C) Back‐spliced junction sequence of circHN1 was validated by Sanger sequencing. (D) qRT‐PCR was performed to detect relative levels of circHN1 and GAPDH after treatment with (+) or without (−) RNase R

CircHN1 is formed by reverse splicing of the linear transcript of exons 3‐5 of *HN1* gene with a length of 260 nucleotides (Figure 2A). PCR and sequencing analyses were conducted to detect the back‐spliced junction sequence of circHN1. The PCR results revealed that only the cDNA fragment could be amplified using circHN1‐specific divergent primers (Figure 2B). The sequencing result showed that the back‐spliced junction sequence of circHN1 was formed by back‐splicing of exon 5 and exon 3 of *HN1* gene (Figure 2C). To further characterize circHN1, RNase R was used to treat total RNA from HGC‐27 cells. The results of PCR showed that circHN1 was more resistant to RNase R digestion compared to linear RNA (Figure 2D). These findings suggest that circHN1 is a circular RNA.

### 3.3 CircHN1 knockdown promotes the proliferation, migration, and invasion of GC cells

**FIGURE 3:**
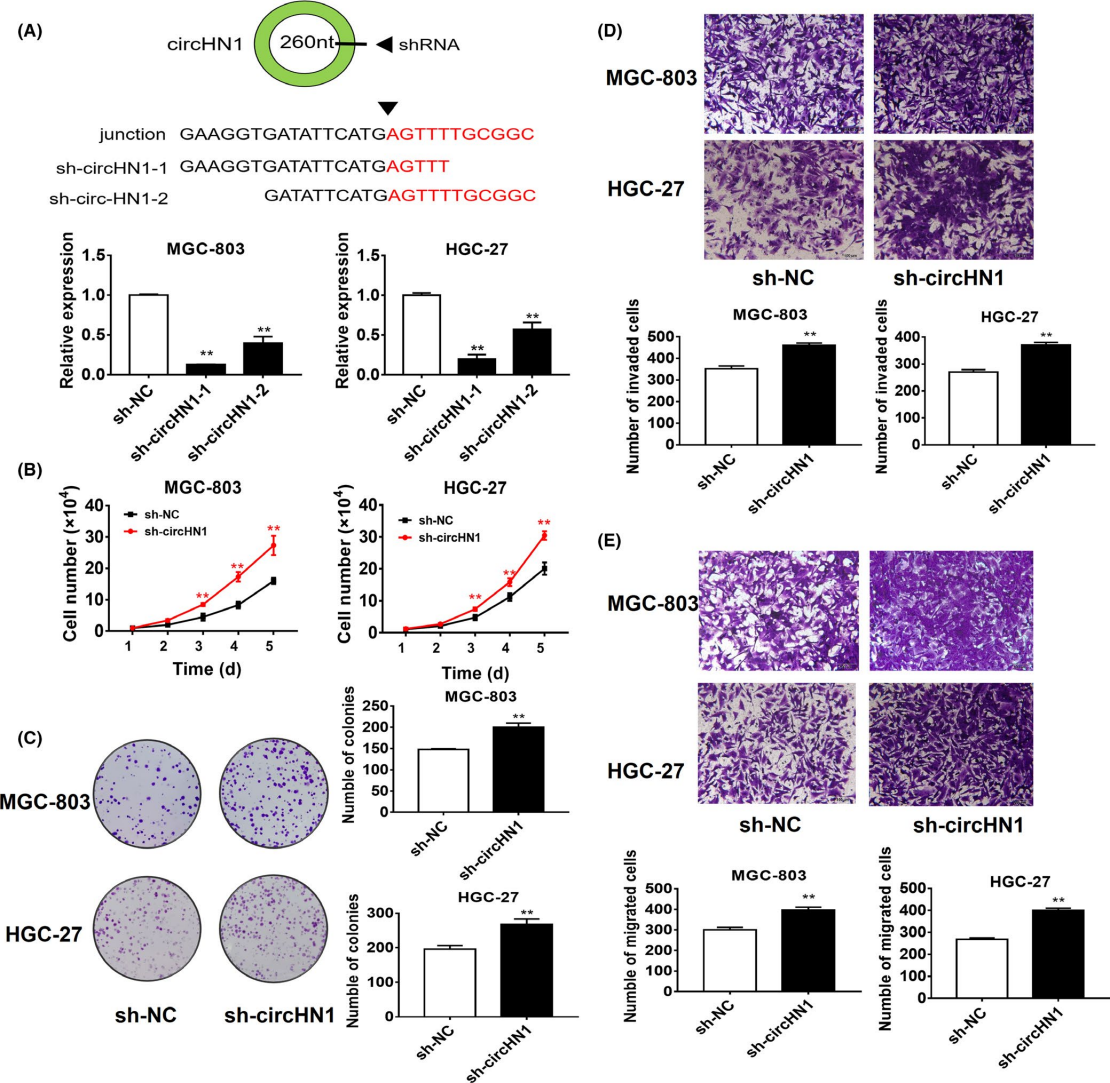
CircHN1 downregulation promotes GC cell proliferation, migration, and invasion. (A) Efficiency of gene knockdown in GC cells by sh‐circHN1 was confirmed by qRT‐PCR. (B) Cell counting, (C) colony formation, (D) transwell migration, and (E) Matrigel invasion assays for circHN1 knockdown and control GC cells

To understand the biological roles of circHN1 in GC progression, we knocked down circHN1 in HGC‐27 and MGC‐803 cells. The knockdown efficiency was verified by RT‐qPCR, and sh‐cirHN1‐1 was chosen for subsequent cell experiments (Figure 3A). The results of cell counting and colony formation assays showed that circHN1 knockdown remarkably increased the proliferation of HGC‐27 and MGC‐803 cells (Figure 3B,3C). We also found that sh‐circHN1 transfection substantially increased the migration abilities of HGC‐27 and MGC‐803 cells compared to that of control cells (Figure 3D). The invasion abilities of HGC‐27 and MGC‐803 cells were also elevated by sh‐circHN1 transfection (Figure 3E).

### 3.4 CircHN1 overexpression inhibits the proliferation, migration, and invasion of GC cells

**FIGURE 4:**
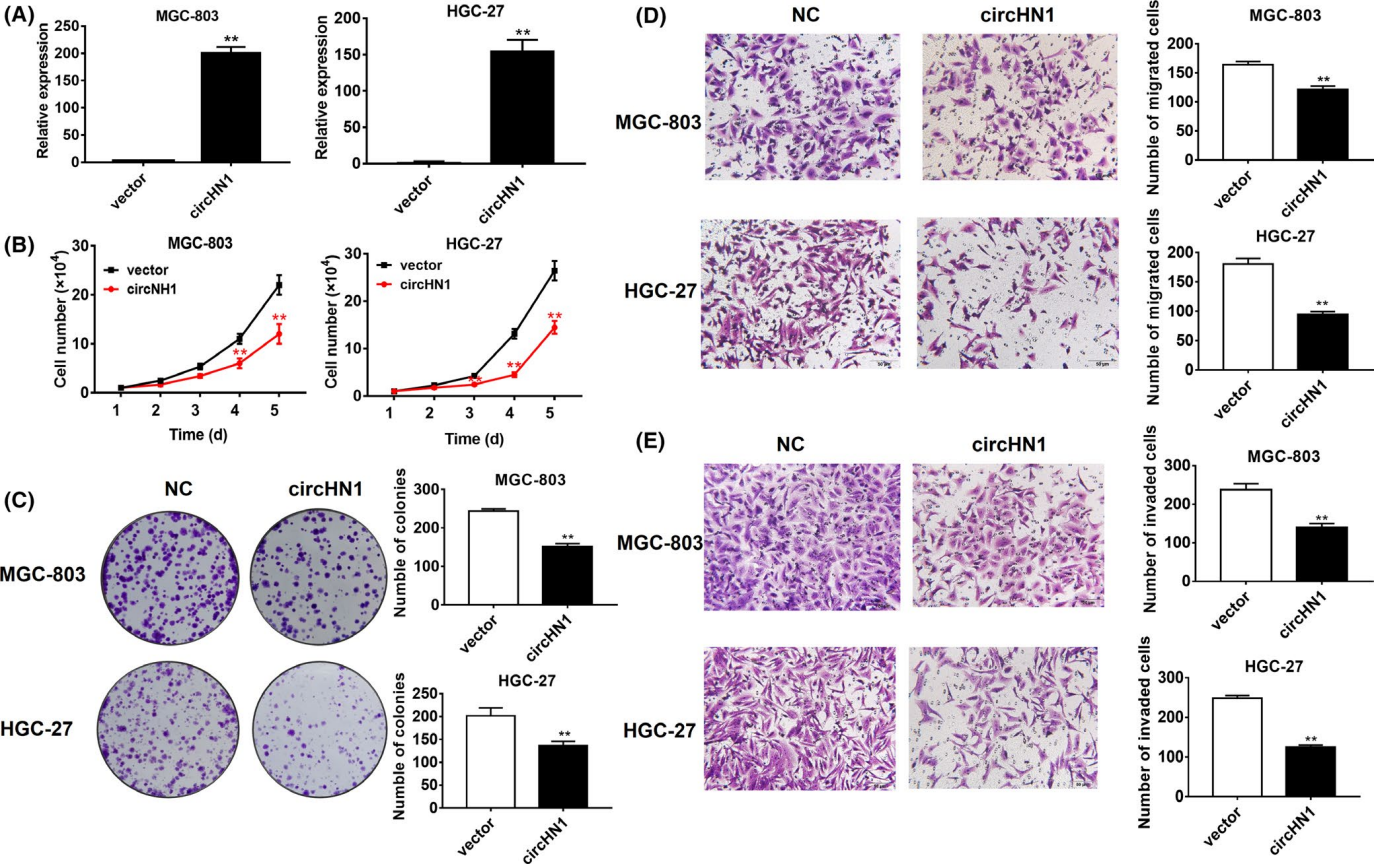
CircHN1 overexpression suppresses GC cell proliferation, migration, and invasion. (A) Efficiency of circHN1 overexpression in GC cells was confirmed by qRT‐PCR. (B) Cell counting, (C) colony formation, (D) transwell migration, and (E) Matrigel invasion assays for circHN1 overexpressing and control GC cells

**FIGURE 5:**
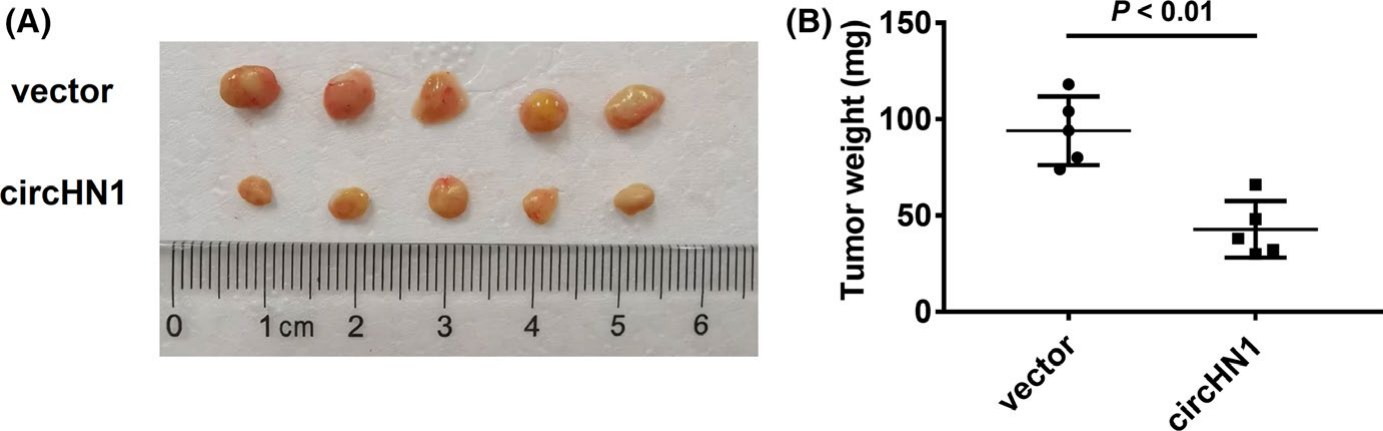
CircHN1 overexpression suppresses GC growth in vivo. (A) Size and (B) weight of xenograft tumors in mice injected with circHN1 overexpressing and control GC cells

We overexpressed circHN1 in HGC‐27 and MGC‐803 cells to further investigate its function (Figure 4A). CircHN1 overexpression remarkably decreased the proliferation (Figure 4B) and colony formation (Figure 4C) of HGC‐27 and MGC‐803 cells. In addition, both the migration (Figure 4D) and invasion (Figure 4E) abilities of HGC‐27 and MGC‐803 cells were inhibited by circHN1 overexpression. We further used a xenograft tumor model to verify the role of circHN1 in vivo. BALB/c nude mice were subcutaneously inoculated with control and circHN1 overexpressing cells. The results showed that both the size (Figure 5A) and weight (Figure 5B) of tumors in the circHN1 group were considerably smaller than those in the control group.

### 3.5 CircHN1 may function as miRNA sponge in GC

**FIGURE 6:**
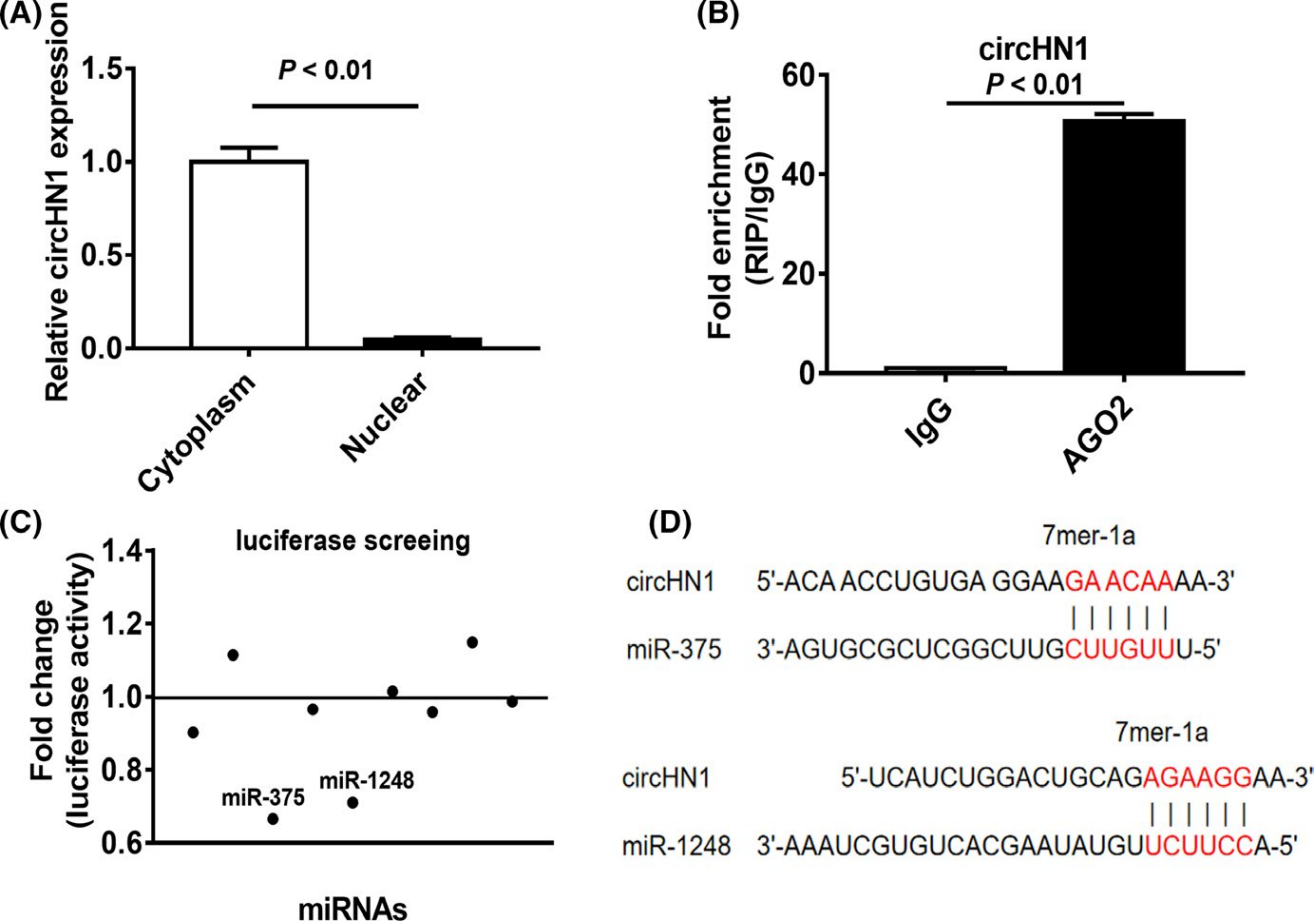
CircHN1 sponges miR‐375 and miR‐1248. (A) Subcellular distribution of circHN1 in GC cells was detected by qRT‐PCR. (B) RIP assay was performed to detect the binding of AGO2 protein to circHN1. (C) Luciferase reporter assays for potential circHN1‐interacting miRNAs. (D) Bioinformatic prediction of the putative binding sites in circHN1 for miR‐375 and miR‐1248

RNA fractionation results indicated that circHN1 was mainly localized in the cytoplasm (Figure 6A). Given that circRNAs can serve as miRNA sponge and circHN1 is abundant in the cytoplasm, we further examined whether circHN1 could bind to specific miRNAs. RIP assay was conducted to analyze the AGO2 occupancy of circHN1. The results revealed that endogenous circHN1 was enriched in the AGO2 immunoprecipitates (Figure 6B), suggesting potential interactions between circHN1 and miRNAs. Using the CircInteractome and StarBase v2.0 miRNA prediction programs, we selected 9 miRNAs (miR‐1184, miR‐1248, miR‐198, miR‐370, miR‐375, miR‐619, miR‐942, miR‐27a, and miR‐27b) which have binding sites for the circHN1 sequence. Dual‐luciferase reporter assays were used to assess which miRNA might bind to circHN1. The circHN1 luciferase reporter plasmid was co‐transfected with different miRNA mimics into 293T cells (Figure 6C). Compared to that in control group, miR‐1248 and miR‐375 inhibited the luciferase reporter activities of circHN1 luciferase reporter (Figure 6C,D). These findings suggest that circHN1 may function as a sponge for these two miRNAs.

## 4 DISCUSSION

The roles of circRNAs in cancer biology have attracted wide attention.^16^ Recent studies have demonstrated that some circRNAs are abnormally expressed in GC and involved in the development and progression of GC.^17^, ^18^, ^19^, ^20^ CircNRIP1 promotes GC progression by sponging miR‐149‐5p and regulating the AKT1/mTOR pathway.^17^ CircFAT1(e2) prevents the development of GC by targeting miR‐548g in the cytoplasm and interacting with the YBX1 protein in the nucleus.^18^ The expression level of hsa_circ_0067582 is significantly downregulated in GC tissues.^19^ Furthermore, hsa_circ_0000419 is lowly expressed in both GC tissues and plasma samples, making it a potential biomarker in GC screening and predictor of patient prognosis.^20^ These results strongly indicate that circRNAs play important roles in the pathophysiology of cancer and may serve as new diagnostic biomarkers and therapeutic targets.

In this study, we identified a new circRNA, circHN1, by using available public circRNA expression profile data for GC. CircHN1 is formed by reverse splicing of the linear transcript of exons 3‐5 of *HN1* gene. HN1 mRNA has been shown to be upregulated in certain cancers.^21^, ^22^, ^23^, ^24^ Increased HN1 expression has been reported to upregulate c‐Met to promote cell growth and migration and is linked to a poor prognosis for patients with hepatocellular carcinoma (HCC).^21^ In breast cancer, HN1 can enhance MYC expression to contribute to cancer progression.^22^ In prostate cancer, HN1 promotes the migration of cancer cells by negatively regulating the interaction of β‐catenin/E‐cadherin.^24^ Our study showed that a circular RNA was generated from this gene locus, suggesting a critical role for *HN1* gene in cancer. However, circHN1 and HN1 mRNA seem to play opposite roles in tumor development.

Generally, highly expressed mRNAs are considered as oncogenes, which affect the development, metastasis, and prognosis of tumors, either directly or indirectly. In our study, we found that circHN1 was overexpressed in GC tissues. However, a high level of circHN1 was negatively associated with tumor invasion. Moreover, circHN1 inhibits the proliferation, migration, and invasion of GC cells in vitro as well as suppresses tumor growth in vivo, which appears contradictory. The difference between mRNAs and circRNAs may explain this inconsistency. For example, it has been reported that cSMARCA5 (circular RNA of SMARCA5) acts as a sponge for miR‐17‐3p and miR‐181b‐5p to inhibit the proliferation and migration of HCC cells, whereas SMARCA5 (mRNA of SMARCA5) activates the Wnt/beta‐catenin signaling pathway to promote HCC proliferation.^25^ In addition, circPOK of *zbtb7a* functions as a proto‐oncogenic RNA by co‐activating the ILF2/3 complex, which operates separately and antithetically with the linear mRNA which serves as a tumor suppressor.^26^ By analyzing the GISTIC and cBioportal databases, we found that the locus of *HN1* was amplified in GC tissues, and HN1 mRNA expression was positively related to the copy number alterations of *HN1* locus, indicating that amplification of its corresponding chromosomal region may cause overexpression of circHN1.

The contradiction between circHN1 expression and function may also be explained by different miRNAs bound by circRNA. One circRNA can bind to multiple miRNAs, and different functions of miRNAs may cause different effects of circRNAs on tumors.^12^ In this study, we found that circHN1 bound to miR‐1248 and miR‐375. MiR‐1248 has been reported to be upregulated in osteosarcoma, and patients with high miR‐1248 expression have poorer survival.^27^ MiR‐1248 can enhance the chemotherapy resistance of osteosarcoma cells by inhibiting AGTR1.^27^ In nephroblastoma, the patients with high expression of miR‐1248 also have poor prognosis.^28^ MiR‐375 plays a dual role in the development and progression of cancer. It acts as an oncomiR in some cancers^29^, ^30^ but as a tumor suppressor miRNA in the others.^31^, ^32^ A high frequency of recurrence and poor survival is observed in GC patients with high level of miR‐375.^33^ MiR‐375 can downregulate the expression of p53 to antagonize ionizing radiation and etoposide treatment in GC cells.^34^ However, other studies have showed that miR‐375 is downregulated in GC and can inhibit the development of gastric cancer by suppressing the Hippo/YAP signaling pathway.^35^ We downloaded the GSE93415 miRNA microarray dataset from the GEO database and analyzed the expression levels of miR‐1248 and miR‐375 in GC tissues. The results showed that miR‐1248 was upregulated in GC tissues (*P* < .01), which is consistent with the upregulation of miR‐1248 expression in various tumors as reported previously.^27^, ^28^ MiR‐1248 may participate in tumor development by promoting tumor cell proliferation and inhibiting apoptosis.^27^ However, miR‐375 was downregulated in GC tissues (*P* < .01). This result is also consistent with that reported in some other studies, suggesting that the oncogenic role of miR‐375 is mainly related to drug resistance, but not to the onset of cancer.^33^, ^34^ Therefore, circHN1 may exert complicated effects on GC development and progression by sponging different miRNAs, including miR‐1248 and miR‐375.

In conclusion, this is the first study to show that circHN1 expression is altered in GC and circHN1 regulates the proliferation and migration of GC cells by sponging miRNAs. CircHN1 may serve as a potential therapeutic target for GC.

## CONFLICT OF INTEREST

The authors declared no conflict of interest.
